# Machine learning-based predictor for neurologic outcomes in patients undergoing extracorporeal cardiopulmonary resuscitation

**DOI:** 10.3389/fcvm.2023.1278374

**Published:** 2023-11-17

**Authors:** Tae Wan Kim, Joonghyun Ahn, Jeong-Am Ryu

**Affiliations:** ^1^ Department of Pulmonary and Critical Care Medicine, Chung-Ang University Hospital, Chung-Ang University College of Medicine, Seoul, Republic of Korea; ^2^ Biomedical Statistics Center, Samsung Medical Center, Data Science Research Institute, Seoul, Republic of Korea; ^3^ Department of Critical Care Medicine, Samsung Medical Center, Sungkyunkwan University School of Medicine, Seoul, Republic of Korea; ^4^ Department of Neurosurgery, Samsung Medical Center, Sungkyunkwan University School of Medicine, Seoul, Republic of Korea

**Keywords:** extracorporeal cardiopulmonary resuscitation, prognosis, machine learning, cerebral performance categories score, extracorporeal membrane oxygenation

## Abstract

**Background:**

We investigated the predictors of poor neurological outcomes in extracorporeal cardiopulmonary resuscitation (ECPR) patients using machine learning (ML) approaches.

**Methods:**

This study was a retrospective, single-center, observational study that included adult patients who underwent ECPR while hospitalized between January 2010 and December 2020. The primary outcome was neurologic status at hospital discharge as assessed by the Cerebral Performance Categories (CPC) score (scores range from 1 to 5). We trained and tested eight ML algorithms for a binary classification task involving the neurological outcomes of survivors after ECPR.

**Results:**

During the study period, 330 patients were finally enrolled in this analysis; 143 (43.3%) had favorable neurological outcomes (CPC score 1 and 2) but 187 (56.7%) did not. From the eight ML algorithms initially considered, we refined our analysis to focus on the three algorithms, eXtreme Gradient Boosting, random forest, and Stochastic Gradient Boosting, that exhibited the highest accuracy. eXtreme Gradient Boosting models exhibited the highest accuracy among all the machine learning algorithms (accuracy: 0.739, area under the curve: 0.837, Kappa: 0.450, sensitivity: 0.700, specificity: 0.740). Across all three ML models, mean blood pressure emerged as the most influential variable, followed by initial serum lactate, and arrest to extracorporeal membrane oxygenation (ECMO) pump-on-time as important predictors in machine learning models for poor neurological outcomes following successful ECPR.

**Conclusions:**

In conclusion, machine learning methods showcased outstanding predictive accuracy for poor neurological outcomes in patients who underwent ECPR.

## Introduction

Neurological prognosis following cardiopulmonary resuscitation (CPR) remains an issue of critical importance for survivors (1, 2). It is important to estimate the potential for normalization of cerebral function in patients after return of spontaneous circulation. The capacity to accurately forecast neurological outcomes can significantly impact subsequent medical management, enabling physicians to make informed decisions that optimize the balance between quality and quantity of intensive treatment (2, 3). Recently, the application of extracorporeal membrane oxygenation (ECMO) as a supplementary measure to conventional CPR has experienced a marked increase (4, 5). Concurrently, the estimation of neurological outcomes for patients subjected to extracorporeal cardiopulmonary resuscitation (ECPR) has become a critical aspect of patient management. However, the task of predicting neurological outcomes post-ECPR is intrinsically complex. It necessitates the comprehensive integration of a myriad of patient-specific factors along with unique circumstances associated with ECPR.

One of the strengths of machine learning (ML) approaches is their capacity to handle intricate nonlinear relationships between predictors, leading to more robust and consistent predictions (6). Harnessing the power of ML could offer a promising solution to the challenge of predicting neurological outcomes after ECPR. This approach can effectively analyze a myriad of patient-specific factors and ECPR-associated circumstances, possibly revealing new correlations and key variables. Consequently, it can enhance the accuracy of neurological prognosis predictions, and direct attention towards the most influential elements impacting patient outcomes in ECPR. While prior studies have identified associations between favorable neurological outcomes and predictors following successful ECPR (7–9), none have explored the potential of machine learning approaches to predict neurological outcomes in ECPR patients. In this study, we aim to utilize ML methodologies to identify critical factors that can influence neurological prognosis following ECPR. We postulate that this innovative approach will shed light on the hidden correlations and interactions among the variables and contribute to a more comprehensive and precise predictive model for neurological outcomes in ECPR patients.

## Methods

### Study population

**Figure 1:**
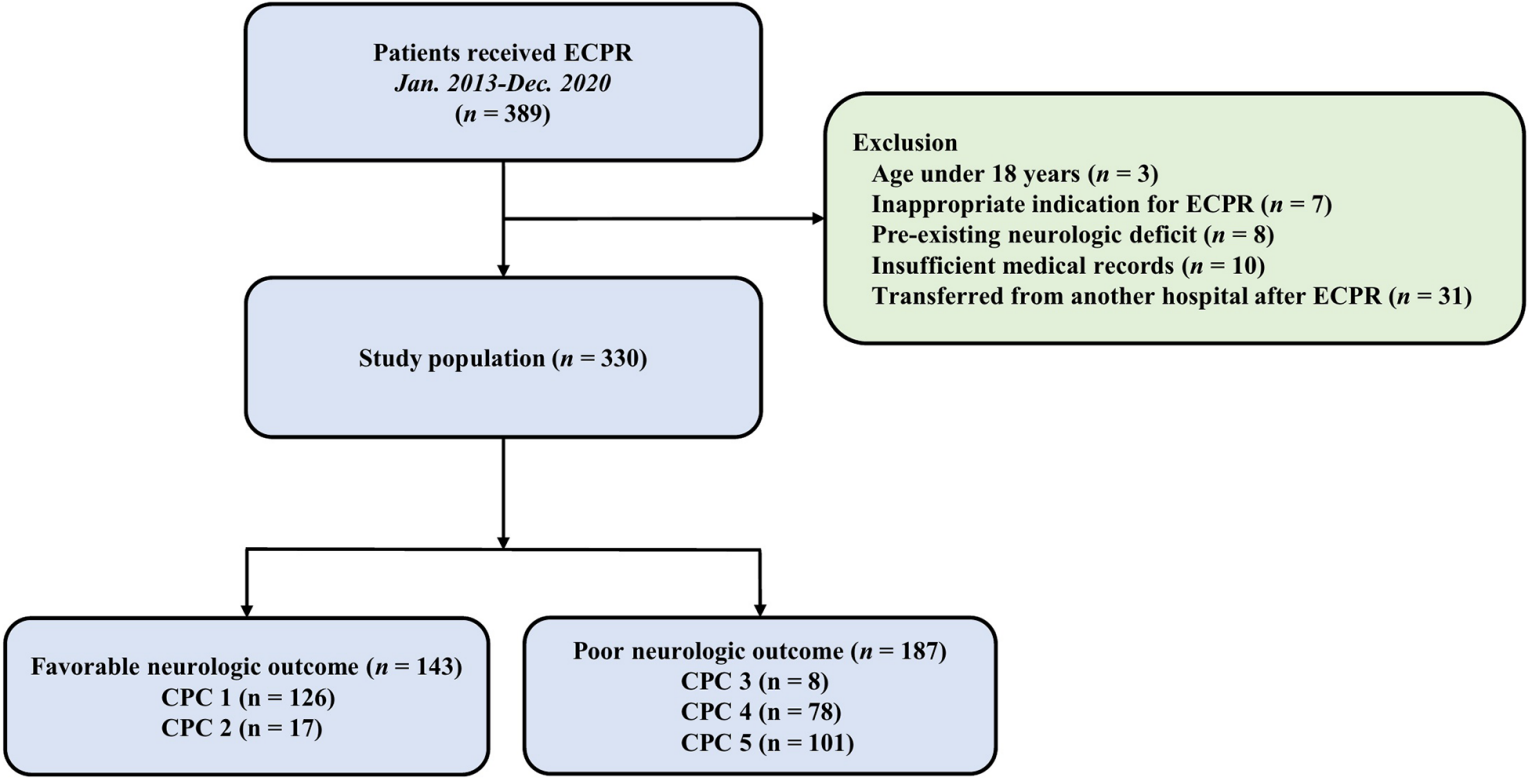
Study flow chart. ECPR, extracorporeal cardiopulmonary resuscitation; CPC, cerebral performance Categories scale.

This study was a retrospective, single-center, observational study that included adult patients who underwent ECPR while hospitalized between January 2010 and December 2020. The Institutional Review Board (IRB) of Samsung Medical Center approved this study (IRB No. 2020-09-082). Informed consent requirements were waived by the Institutional Review Board (IRB) of Samsung Medical Center, given the retrospective nature of the study. The study included all consecutive patients who underwent ECPR during the study period, resulting in a total of 389 patients. Of these patients who under the age of 18, those with inappropriate indications for ECPR, those with pre-existing severe neurological conditions such as traumatic brain injury, major stroke, malignant brain tumor, or severe dementia, those with insufficient medical records, and those who were transferred from another hospital after undergoing ECPR were excluded (Figure 1).

### Definitions and outcomes

In this study, we retrospectively collected baseline characteristics, including comorbidities, behavioral risk factors, intensive care unit management, and laboratory data, utilizing our center’s dedicated “Clinical Data Warehouse Darwin-C.” This data warehouse has been specifically designed to facilitate investigators in searching and retrieving de-identified medical records from electronic archives. Mean blood pressure (MBP) was the mean of the values measured in the first 24 h, mainly based on arterial blood pressure, and patients without ABP used non-invasive blood pressure instead. Laboratory data was characterized by the most unfavorable value recorded within the 6 h window immediately preceding ECMO insertion, inclusive of the period during CPR. In this study, ECPR was defined as both a successful veno-arterial ECMO implantation and pump-on with cardiac massage during the index procedure in patients with cardiac arrest. Importantly, when ROSC occurs during ECMO cannulation, practitioners generally do not remove the already-inserted cannula nor do they halt the ECMO pump activation process, as referenced in studies (1, 10). The term “ECMO pump-on” was characterized as the cessation of chest compressions following the successful implantation and activation of the ECMO device. In this study, ECPR was initiated under specific criteria: a witnessed arrest was confirmed; conventional CPR had been administered for a duration exceeding 10 min without success; and the etiological event causing the cardiac arrest was deemed reversible (4). Exceptions to ECPR initiation were cases with: anticipated life expectancy of less than 6 months; terminal malignancy; an unwitnessed collapse; limited physical activity; an unprotected airway; or instances where CPR had been performed for over 60 min at the time of initial contact. It should be noted that age alone was not considered a contraindication for the initiation of ECPR (4). ECPR was defined as use of venoarterial ECMO intended to treat cardiac arrest and arrest to ECMO pump-on time was defined as time from collapse to the point of ECMO setup and administration (11). In patients undergoing ECPR, the process of extracorporeal circulation, combined with external volume infusion, has the potential to decrease body temperature. This reduction in temperature could confer some degree of neuroprotection via induced hypothermia. It should be noted that aggressive therapeutic hypothermia might not always be pursued in cases where the patient exhibits hemodynamic instability or has complications such as bleeding during ECMO support. Consequently, in the context of ECPR, the initiation and extent of surface cooling, as well as the targeted temperature, are individually determined by the attending ICU intensivist. This decision-making process adheres to the therapeutic hypothermia protocol established by Samsung Medical Center (12). The primary outcome was neurologic status at hospital discharge as assessed by the Glasgow-Pittsburgh Cerebral Performance Categories (CPC) score (scores range from 1 to 5) (13). CPC scores of 1 and 2 were classified as favorable neurologic outcomes; CPC scores of 3, 4, and 5 were considered poor neurologic outcomes (14, 15). We thoroughly reviewed medical records and patients were assigned to the CPC scale upon agreement by two authors (JAR and TWK).

### Machine learning (Ml) models

Utilizing Shapley Additive exPlanations, we first identified the critical variables, which were then incorporated into the ML analyses. Initially, we trained and tested eight ML algorithms for a binary classification task involving the neurological outcomes of survivors after ECPR. The algorithms included logistic regression (LR), random forest (RF), AdaBoost Classification Trees (AdaBoost), Bagged CART (Bagging), Stochastic Gradient Boosting (GBM), eXtreme Gradient Boosting (XGBoost), Multivariate Adaptive Regression Spline (MARS), and Support Vector Machines with Radial Basis Function Kernel (SVM). For the final analysis, we only utilized the top three algorithms with the highest accuracy out of the aforementioned eight ML algorithms. We divided the dataset into training and testing sets with an 8:2 ratio. The training set was used for statistical analysis, feature selection, and model training, while the independent testing set was employed to evaluate the trained models. Additionally, we detected a small number of missing values in the dataset. To address this issue, we utilized the k-Nearest Neighbors algorithm for imputation during the ML analysis (16–18). This technique involved estimating the missing values by considering the values of their nearest neighbors in the dataset. By applying this approach, we ensured that the dataset was complete and ready for further analysis and modeling. Furthermore, preprocessing procedure entailed scaling and one-hot encoding. Due to the limited sample size, we opted for the Leave-One-Out Cross-Validation (LOOCV) methodology. This approach could minimize bias by assessing the algorithm across the entire dataset, thereby ensuring more consistent and reproducible results. Afterwards, we trained each ML algorithm using the best hyperparameters until convergence was achieved on the training set. The cutoff threshold for each model was determined based on the receiver operating characteristic curve and Youden index (19, 20) obtained from the validation set, and this threshold was then applied to the test set. In order to identify which variables have the predictive performance, the importance of each variable in the ML model was evaluated by the permutation score of the test set. This score is defined as a decrease in model performance (area under the receiver operating characteristic curve) when all values of a given variable are randomly mixed (21). The magnitude of the model performance reduction reflects how dependent the model is on particular variable. The importance of variables is scaled so that the maximum value is 100.

### Statistical analyses

For continuous variables, we first assessed their distribution for normality. Variables that followed a normal distribution were presented as means ± standard deviations, while those that did not were described using medians and interquartile ranges. Categorical variables are represented as numbers with subsequent percentages. Data comparison was carried out using Student’s *t*-test or Mann-Whitney *U* test for continuous variables, whereas the Chi-square test for categorical variables. Clinically relevant variables, including age, sex, comorbidities, habitual risk factors, variables associated with ECPR, classification of arrest subtypes, complications of ECMO, MBP and ICU management were subjected to multiple logistic regression analyses to obtain statistically meaningful predictors associated with poor neurological outcomes. All tests were two-sided and *p* values of less than 0.05 were considered statistically significant. Statistical analyses were performed with R Statistical Software (version 4.2.0; R Foundation for Statistical Computing, Vienna, Austria).

## Results

### Baseline characteristics and clinical outcomes

During the study period, 330 patients were finally enrolled in this analysis; 143 (43.3%) had favorable neurological outcomes but 187 (56.7%) did not. The characteristics of the patients are shown in Table 1. There was no difference between the two groups in the age, sex, and comorbidities except for chronic kidney disease. Compared to the group with poor neurological outcomes, the group with favorable outcomes exhibited a higher prevalence of shockable rhythms, ECPR in the coronary catheterization laboratory, cardiac cause of arrest, and arrest by acute coronary syndrome. Hemoglobin was also higher in favorable group than poor group (10.6 ± 2.6 g/dl vs. 9.6 ± 3.0 g/dl, *p *= 0.001), but troponin I was no significant difference between two groups (0.7 [0.1–4.0] ng/ml vs. 0.9 [0.1–7.2] ng/ml, *p *= 0.211). Arrest to ECMO pump-on time (23.0 [11.0–35.0] min vs. 35.0 [24.5–49.5] min, *p *< 0.001) and serum lactate (7.9 [5.5–12.9] mmol/L vs. 12.5 [9.2–15.0] mmol/L, *p *< 0.001) were higher in the poor neurologic outcome group than in the favorable group.

**Table 1:** Baseline characteristics of patients.

	Favorable neurologic outcome (*n* = 143)	Poor neurologic outcome (*n* = 187)	*P* value
Patient demographics			
Age, years	60.0 [49.0–68.0]	62.0 [50.5–72.5]	0.100
Sex, male	103 (72.0)	131 (70.1)	0.788
Comorbidities			
Malignancy	20 (14.0)	37 (19.8)	0.217
Hypertension	60 (42.0)	94 (50.3)	0.165
Diabetes mellitus	44 (30.8)	65 (34.8)	0.519
Chronic kidney disease^a^	9 (6.3)	28 (15.0)	0.021
Cardiovascular disease	35 (24.5)	43 (23.0)	0.855
Stroke	16 (11.2)	12 (6.4)	0.180
CPR details			
Type of cardiac arrest			0.032
Out of hospital cardiac arrest	13 (9.1)	32 (17.1)	
In-hospital cardiac arrest	131 (91.6)	155 (82.9)	
First monitored rhythm			0.002
Asystole	14 (9.8)	38 (20.3)	
Pulseless electrical activity	71 (49.7)	104 (55.6)	
Shockable rhythm (VT or VF)	57 (39.9)	42 (22.5)	
Arrest to ECMO pump-on time, minutes	23.0 [11.0–35.0]	35.0 [24.5–49.5]	<0.001
Cause of CPR			
Cardiac cause	109 (76.2)	97 (51.9)	<0.001
Acute coronary syndrome	87 (60.8)	72 (38.5)	
Dilated Cardiomyopathy	5 (3.5)	9 (4.8)	
Myocarditis	8 (5.6)	4 (2.1)	
Refractory arrhythmia	4 (2.8)	3 (1.6)	
Stress-induced cardiomyopathy	2 (1.4)	5 (2.7)	
Heart transplant rejection	3 (2.1)	1 (0.5)	
Valvular heart disease	0 (0)	3 (1.6)	
Non-cardiac cause	34 (23.8)	90 (48.1)	0.003
Respiratory failure	6 (4.2)	18 (9.6)	
Pulmonary embolism	5 (3.5)	17 (9.1)	
Aortic syndrome	4 (2.8)	9 (4.8)	
Sepsis	2 (1.4)	10 (5.3)	
Non-traumatic bleeding	2 (1.4)	6 (3.2)	
Trauma	2 (1.4)	4 (2.1)	
Others	13 (9.1)	26 (13.9)	
Location of ECPR			0.006
Intensive care unit	38 (28.1)	52 (28.6)	
Emergency department	36 (26.7)	62 (34.1)	
Coronary catheterization lab	31 (23.0)	15 (8.2)	
General ward	27 (20.0)	48 (26.4)	
Operation room	3 (2.2)	5 (2.7)	
Anoxic brain injury^b^	7 (4.9)	78 (41.7)	<0.001
Mean blood pressure	75.1 [67.8–80.9]	64.8 [56.3–76.5]	<0.001
Laboratory data			
Hemoglobin, g/dl	10.6 ± 2.6	9.6 ± 3.0	0.001
Platelet, ×10^3^ /μl	194.0 [123.0–258.0]	183.0 [103.0–242.0]	0.109
Total bilirubin, mg/dl	0.8 [0.4–1.3]	0.9 [0.5–1.6]	0.162
Blood urea nitrogen, mg/dl	17.2 [12.7–23.4]	23.1 [16.3–34.1]	<0.001
Creatinine, mg/dl	1.1 [0.8–1.5]	1.4 [1.0–2.0]	<0.001
Troponin I	0.7 [0.1–4.0]	0.9 [0.1–7.2]	0.211
Lactate	7.9 [5.5–12.9]	12.5 [9.2–15.0]	<0.001
Arterial blood gas analysis			
pH	7.3 [7.2–7.4]	7.3 [7.1–7.4]	<0.001
PaO_2_	89.9 [65.2–119.8]	79.1 [47.5–125.2]	0.049
PaCO_2_	34.0 [29.0–45.9]	42.4 [32.5–65.7]	<0.001

Data are presented as mean ± standard deviation, median (interquartile range), or *n* (%).

CPR, cardiopulmonary resuscitation; ECMO, extracorporeal membrane oxygenation; ECPR, extracorporeal cardiopulmonary resuscitation; VF, ventricular fibrillation; VT, ventricular tachycardia.

^a^ Chronic kidney disease is defined as either kidney damage or glomerular filtration rate less than 60 ml/min/1.73 m^2^ for 3 months or longer.

^b^ Anoxic brain injury defined as presence of a marked deduction of the gray-white ratio on brain CT (<1.1) (22)or extensive restriction of diffusion on brain MRI (23).

In multivariate analysis, age (adjusted odds ratio [OR]: 2.18, 95% confidence interval [CI]: 1.56–3.13), chronic kidney disease (adjusted OR: 1.53, 95% CI: 1.10–2.19), ECPR in the coronary catheterization laboratory (adjusted OR: 0.61, 95% CI: 0.43–0.85), cardiac cause of arrest (adjusted OR: 0.56, 95% CI: 0.39–0.78), arrest to ECMO pump-on time (adjusted OR: 1.59, 95% CI: 1.10–2.41), PaCO_2_ (adjusted OR: 1.84, 95% CI: 1.25–2.83), initial hemoglobin (adjusted OR: 0.67, 95% CI: 0.47–0.98), and MBP (adjusted OR: 0.43, 95% CI: 0.29–0.61) were associated with poor neurological outcomes.

### ML-based predictive performance of poor neurologic outcome after ECPR

**Figure 2:**
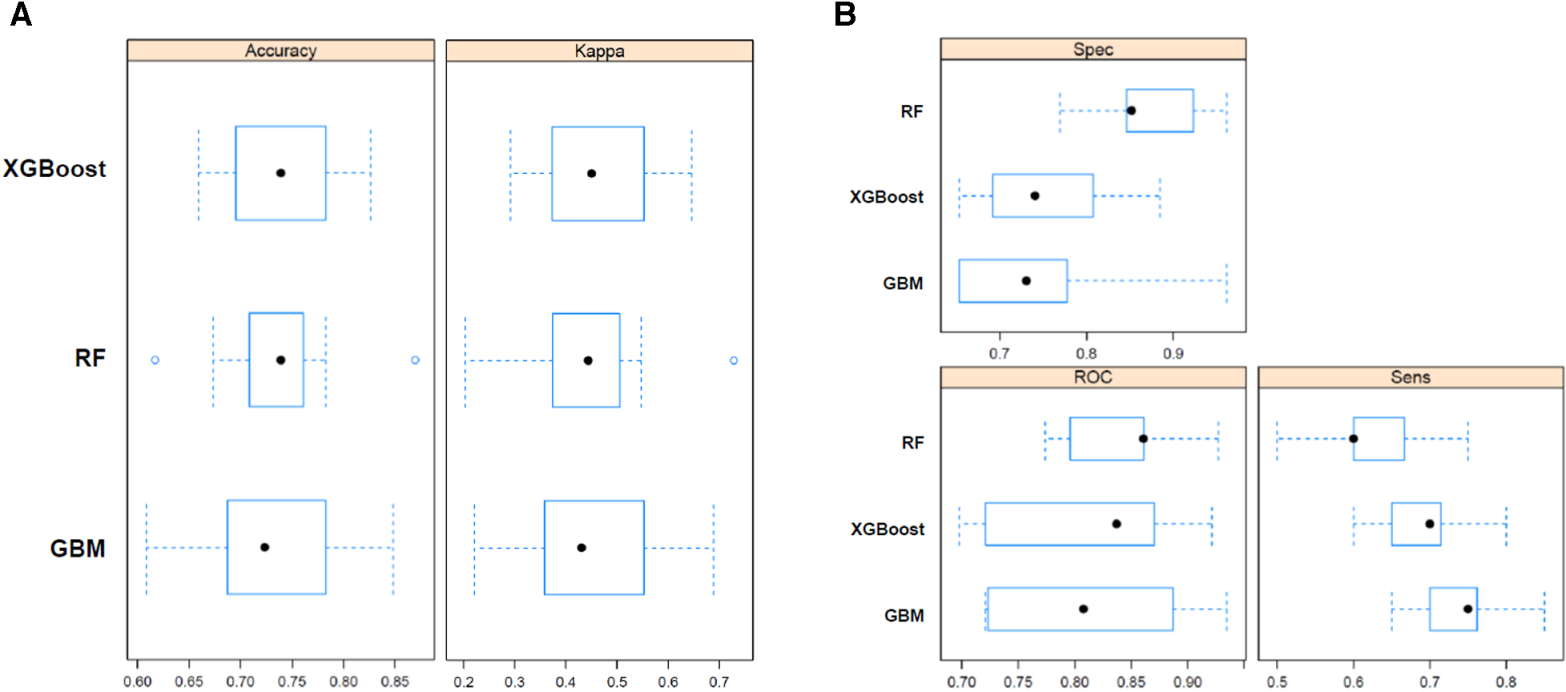
Predictive performance of random forest (RF), bagged CART (bagging), and eXtreme gradient boosting (XGBoost) machine learning model for poor neurologic outcome. Sens, sensitivity; Spec, specificity; ROC, receiver operating characteristic.

**Figure 3:**
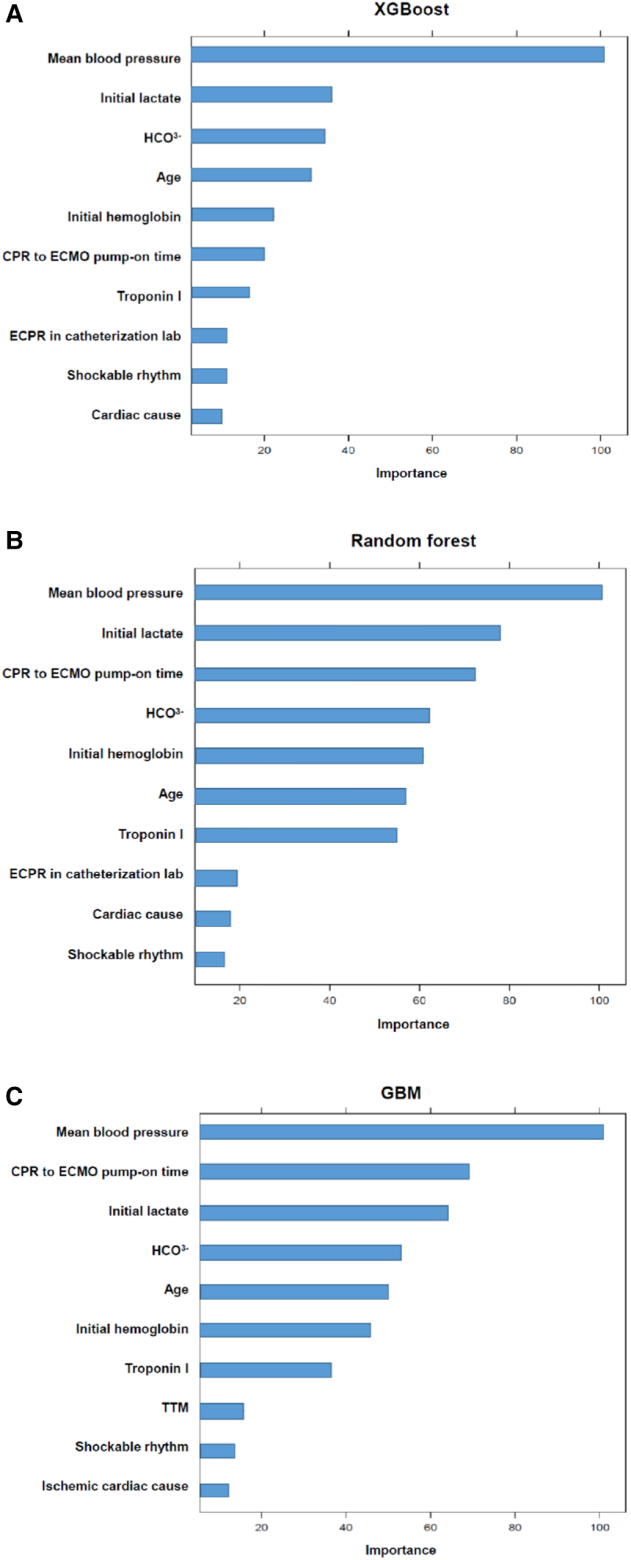
The top variables contributing to the predictive performance of each model. The magnitude of the model performance reduction reflects how dependent the model is on particular variable. The importance of variables is scaled so that the maximum value is 100.

The predictive performances of all algorithms were depicted in Supplementary Figure S1. After initial analysis using ML models, LR, AdaBoost, Bagging, MARS, and SVM were excluded in final analysis because of relatively low predictive power. We only utilized the top three algorithms, XGBoost, RF, GBM, and with the highest accuracy from the eight ML algorithms. Predictive performance of each ML model for poor neurologic outcome was shown in Figure 2 and Table 2. Overall, all three models showcased excellent proficiency in predicting poor neurological outcomes, with mean accuracy scores ranging between 72.3% and 73.9%. Notably, XGBoost models exhibited the highest accuracy among all the machine learning algorithms (Table 3). Figure 3 illustrated the top 10 variables that contribute to the predictive performance of each ML model. Across all three ML models, MBP emerged as the most influential variable, followed by initial serum lactate, and arrest to ECMO pump-on-time as important predictors. Finally, we tested the XGBoost model using the testing dataset, and it exhibited excellent predictive performance for poor neurological outcomes (accuracy: 0.712, 95% CI: 0.609–0.809, Kappa: 0.213, sensitivity: 0.643, specificity: 0.771, positive predictive value: 0.667, negative predictive value: 0.546). Additionally, the performance of initial lactate level for prediction of poor neurologic outcomes was evaluated. The area under the receiver operating characteristic curve was 0.66 (95% CI: 0.598–0.724) and the cut-off value was 7.37 with 86.6% sensitivity and 46.4% specificity.

**Table 2:** Clinical outcomes according to neurologic outcomes.

	Favorable neurologic outcome (*n* = 143)	Poor neurologic outcome (*n* = 187)	*P* value
ICU management			
ECMO duration, hours	61.1 [24.1–123.0]	52.5 [11.0–118.1]	0.117
Targeted temperature management^a^	12 (8.4)	32 (17.1)	0.032
CRRT	42 (29.4)	92 (49.2)	<0.001
Intra-aortic balloon pump	10 (7.0)	8 (4.3)	0.406
Mechanical ventilation	109 (76.2%)	149 (79.7%)	0.536
Limb ischemia	8 (5.6)	13 (7.0)	0.785
ECMO site bleeding	16 (11.2)	22 (11.8)	0.999
Stroke after ECPR	7 (4.9)	12 (6.4)	0.727
Gastrointestinal bleeding	1 (0.7)	7 (3.8)	0.167
Sepsis	0 (0.0)	6 (3.2)	0.081
Rhabdomyolysis	6 (4.2)	5 (2.7)	0.650
Clinical outcomes			
In-hospital mortality	0 (0.0)	101 (54.0)	<0.001
Hospital length of stay, days	21.0 [12.0–55.5]	4.0 [1.0–13.0]	<0.001

Data are presented as mean ± standard deviation, median (interquartile range), or *n* (%).

CRRT, continuous renal replacement therapy; ECMO, extracorporeal membrane oxygenation; ICU, intensive care unit.

^a^ Targeted temperature management was performed by using surface cooling device (Arctic Sun).

**Table 3:** Model performance in predicting poor neurologic outcome after extracorporeal cardiopulmonary resuscitation.

Algorithm	Accuracy	AUC	Sensitivity	Specificity	Kappa
XGboost	0.739	0.837	0.700	0.740	0.450
RF	0.734	0.860	0.600	0.851	0.443
GBM	0.723	0.807	0.750	0.730	0.430

AUC, area under the curve; GBM, stochastic gradient boosting; RF, random forest; XGBoost, eXtreme gradient boosting.

## Discussion

In the present study, we investigated the predictors of poor neurological outcomes in ECPR patients using ML approaches. From the eight ML algorithms initially considered, we refined our analysis to focus on the three algorithms, XGBoost, RF and GBM, that exhibited the highest accuracy. XGBoost models exhibited the highest accuracy among all the machine learning algorithms. In addition, when we tested the XGBoost model using the testing dataset, it demonstrated outstanding predictive accuracy for poor neurological outcomes. Across all three ML models, MBP emerged as the most influential variable, followed by initial serum lactate, and arrest to ECMO pump-on-time as important predictors.

Generally, lactic acid serves as a valuable indicator of tissue hypoxia (24) and is a reliable predictor of patient outcomes in cases of circulatory shock (24–26). Previous studies showed that serum lactic acid levels are associated with neurological outcomes in survivors after cardiac arrest. Sawamoto et al. demonstrated a significant difference in serum lactic acid levels between patients with favorable and poor neurological outcomes who underwent ECPR (27). Moreover, Christian et al. demonstrated that absolute serum lactate levels might serve as pertinent markers for predicting mortality in ECPR patients. Furthermore, lactate clearance was associated with neurological outcomes in these patients (28). The findings from this study highlighted that the serum lactate level served as a prognostic indicator for poor neurological outcomes in patients treated with ECPR across all ML models.

Brain recovery hinges on the swift restoration of cerebral blood flow to meet the brain’s metabolic demands, with MAP being a principal determinant of this flow (29). The current guideline recommends circumvention and immediate correction of MAP less than 65 mmHg in post-resuscitation care (23). However, the exact MAP target conducive to optimal outcomes remains elusive, and several studies have indicated a potential correlation between higher MAP values and more favorable neurological results (30, 31). Specifically, Lee et al. found that average MAP levels were associated with neurological outcomes in patients undergoing ECPR (32). They suggest that maintaining an average MAP of approximately 75 mmHg could be pivotal for neurological recovery after ECPR. Our study also highlighted that the MAP served as a prognostic indicator for poor neurological outcomes in patients treated with ECPR across all ML models.

Given that the brain is the organ most susceptible to hypoxia and insufficient perfusion, delays in initiating ECMO during ECPR can lead to significant neurological deficits (33). Several previous studies have demonstrated that duration of no flow or low-flow is one of the most important predictors of overall outcomes after ECPR along with factors such as age, initial shockable rhythm and lactate level (4, 34, 35). Recently, Matsuyama et al. analyzed 256 patients undergoing ECPR and found the probability of favorable neurological outcome decreased as low-flow duration increased. Similarly, low-flow time represented by arrest to pump-on time was associated with poor neurologic outcomes in the present study. Eventually, enhancing survival and neurological outcomes is more likely when patients are put on the ECMO pump-on promptly (4, 34, 36).

In our previous study, factors such as shockable rhythm, initial hemoglobin levels, cardiac cause of arrest and ECPR conducted at the cardiac catheterization lab were identified as significantly associated with poor neurological outcomes (2, 4, 37). Low pre-ECMO hemoglobin levels might correlate with adverse neurological results (4, 28). Proactively addressing anemia either before or during ECMO deployment may improve oxygen delivery and offer neuroprotection (4). Shockable rhythm was associated with favorable neurological outcomes after ECPR (2, 38). ECPR conducted in a cardiac catheterization lab resulted in a reduction of low flow and cannulation time (39). In this study, most of the patients with cardiac arrest in the catheterization lab underwent ECPR in the coronary catheterization laboratory. Reducing “arrest to ECMO pump-on time” would be crucial to improve clinical outcomes, including neurologic outcomes, regardless of the location of ECPR.

This study had several limitations. First, this was a nonrandomized cohort study. Therefore, confounding factors and selection bias might have affected the results. Second, CPC scale was retrospectively determined based on medical records. We excluded patients whose neurological status could not be assessed because of deterioration followed by death. However, we included patients who had a diagnosis of brain death. Third, most ECPR patients had low body temperature caused by extracorporeal circulation and external volume infusion. Therefore, ECMO itself could have some degree of neuroprotective effect through hypothermia. Finally, lactate clearance is associated with neurological outcomes in ECPR patients, but due to the nature of retrospective studies, it cannot be provided due to insufficient data after a specific time following the initial lactic acid test.

## Conclusions

In conclusion, serum lactic acid levels and arrest to ECMO pump-on time emerged as the most potent predictors in machine learning models for poor neurological outcomes following successful ECPR. Furthermore, these machine learning methods showcased outstanding predictive accuracy for poor neurological outcomes in patients who underwent ECPR.

## Data Availability

The raw data supporting the conclusions of this article will be made available by the authors, without undue reservation.
