# Supplementary material for: Machine learning-based predictor for neurologic outcomes in patients undergoing extracorporeal cardiopulmonary resuscitation
Source: Front Cardiovasc Med. 2023 Nov 17;10:1278374. doi: 10.3389/fcvm.2023.1278374 (PMC10691482; doi:10.3389/fcvm.2023.1278374)

**Figure S1.** Predictive performance of eight machine learning model for poor neurologic outcome. AdaBoost, AdaBoost Classification Trees; Bagging, Bagged CART; LR, logistic regression; MARS, Multivariate Adaptive Regression Spline; RF, random forest; GBM, Stochastic Gradient Boosting; SVM, Support Vector Machines with Radial Basis Function Kernel; XGBoost, eXtreme Gradient Boosting.
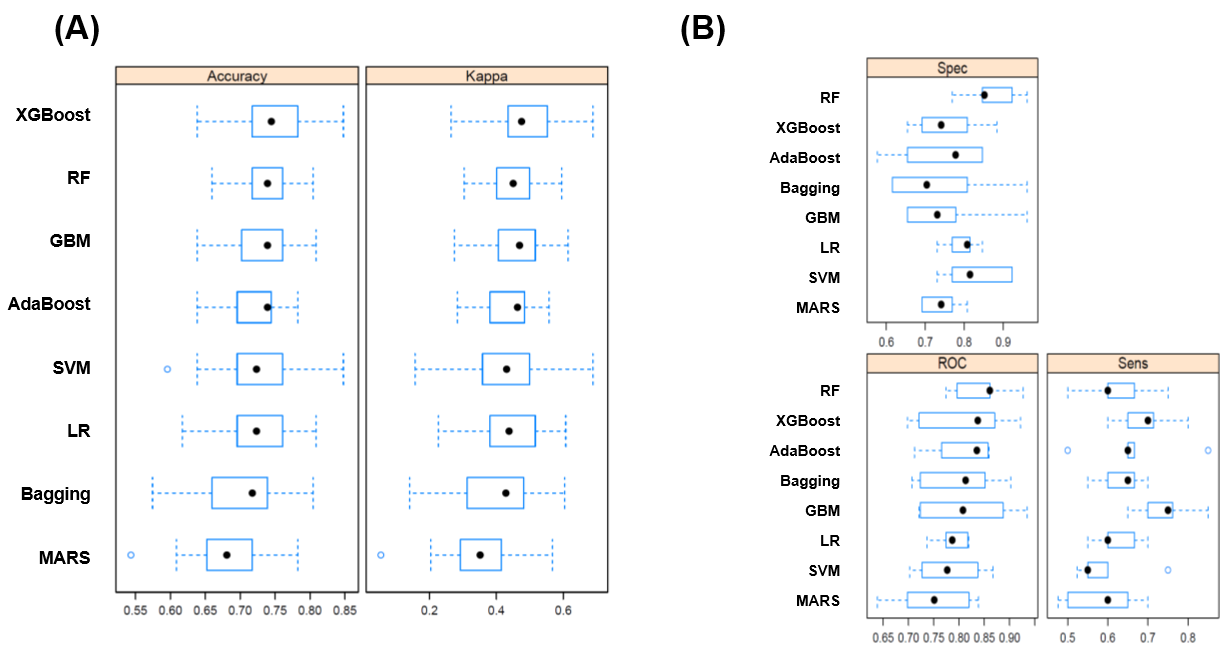

Supplement: Supplementary file 1 [file Datasheet1.docx]
